# NRAS Mutations in Different Melanoma Histotypes: Molecular and Clinical Implications

**DOI:** 10.1007/s11912-026-01851-x

**Published:** 2026-10-26

**Authors:** Marina Pisano, Silvia Tore, Sara Demartis, Roberto Nico Dallocchio, Stefania Casula, Maria Colombino, Antonella Manca, Manuela Usai, Luca Nuvoli, Giuseppe Palmieri, Milena Casula

**Affiliations:** 1 Istituto di Ricerca Genetica e Biomedica, Consiglio Nazionale delle Ricerche, Traversa La Crucca 3, Sassari, 07100 Italy; 2 Istituto di Chimica Biomolecolare, Consiglio Nazionale delle Ricerche, Traversa La Crucca 3, Sassari, 07100 Italy; 3 Dipartimento di Medicina, Chirurgia e Farmacia, Università degli Studi di Sassari, Viale San Pietro 43, Sassari, 07100 Italy

**Keywords:** Melanoma, NRAS mutations, Histotype-specific profiles, Tumor heterogeneity, Target therapy and Immunotherapy, Precision oncology

## Abstract

**Purpose of Review:**

Melanoma is a highly aggressive skin malignancy with increasing incidence and mortality worldwide. Among its key genetic alterations, NRAS mutations occur in approximately 15–25% of melanomas, defining a distinct molecular subset associated with aggressive tumor behavior and poor prognosis. Unlike BRAF mutations, NRAS alterations remain largely undruggable, posing significant therapeutic challenges. This review examines the histotype-specific distribution of NRAS mutations in melanoma and discusses their molecular and clinical implications.

**Recent Findings:**

Most NRAS mutations involve codon 61 and result in constitutive activation of the MAPK and PI3K signaling pathways, thereby promoting tumorigenesis and disease progression. Although NRAS-mutant melanomas are associated with an immunosuppressive tumor microenvironment, they also exhibit high immunogenicity, suggesting potential sensitivity to immune checkpoint inhibitors (ICIs). To further characterize the molecular landscape of NRAS-mutant melanoma, we analyzed data from the Catalogue of Somatic Mutations in Cancer (COSMIC), providing insights into the prevalence, distribution, co-mutation patterns, and functional consequences of NRAS alterations across different melanoma histotypes.

**Summary:**

NRAS mutations represent a major driver of melanoma progression and define a biologically and clinically distinct subset of the disease. Despite encouraging responses to immunotherapy, clinical outcomes remain suboptimal, highlighting the need for novel therapeutic approaches. A deeper understanding of NRAS-driven oncogenesis, histotype-specific mutation patterns, and their impact on therapeutic response is essential for advancing precision medicine and improving outcomes for patients with melanoma.

## Introduction

Malignant Melanoma (MM) is an aggressive skin cancer originating from melanocytes and is among the deadliest malignancies worldwide. Its incidence has steadily risen, with over 330,000 new cases and approximately 59,000 deaths reported globally in 2022, particularly for Caucasian populations in regions with high ultraviolet (UV) exposure, such as Australia and New Zealand [1]. Over the past two decades, molecular studies have greatly improved our understanding of melanoma, especially its genetic and molecular features. A hallmark of this malignancy is the dysregulation of the mitogen-activated protein kinase (MAPK) pathway, a critical regulator of cellular proliferation, differentiation, and survival. Among all cancers, melanoma exhibits one of the highest burdens of genetic mutations [2]. According to The Cancer Genome Atlas (TCGA) [3], mutations in BRAF, NRAS, and NF1 are the most frequent genetic alterations in melanoma. These mutations drive persistent activation of the MAPK pathway, which is pivotal in melanoma initiation and progression. The identification of driver mutations such as BRAFV600, present in 40–50% of melanomas [4, 5], has revolutionized treatment paradigms, enabling the development of targeted therapies like BRAF and MEK inhibitors, as well as immunotherapies targeting PD-1/PD-L1 and CTLA-4 [6, 7]. However, the intrinsic heterogeneity of metastatic melanoma continues to pose significant challenges. This variability, observed at both inter- and intra-tumor levels, impacts treatment response and resistance mechanisms [8, 9]. Advances in Next-Generation Sequencing (NGS) have further illuminated the molecular complexity of melanoma, uncovering mutations in genes such as BRAF, NRAS, TP53, CDKN2A, PTEN, MAP2K1-2, KIT, and NF1, alongside recurrent copy number variations (CNVs) such as CCND1 amplification and CDKN2A deletions [10]. These findings underscore the intricate interplay of genetic and epigenetic alterations in melanoma progression.

### Oncogenic Role of NRAS in Melanoma

### NRAS Mutations Spectrum and Clinical Relevance

Despite being less common than BRAF mutations, NRAS mutations are clinically significant, occurring in an estimated rate of 75,000 melanoma patients annually, covering 16–25% of disease diagnoses [11, 12]. Progression-free survival (PFS) in NRAS-mutant melanoma is generally shorter than in BRAF-mutant cases [13]. Unlike BRAF mutations, which are common in benign nevi, NRAS mutations are rare in benign lesions except for congenital nevi and tend to occur more frequently in older patients [14–16]. While BRAF mutations are more prevalent in melanomas arising in intermittently sun-exposed areas, NRAS mutations are observed in both chronic sun-damaged (CSD) and non-CSD melanomas, with a noted predilection for the upper extremities [17, 18]. NRAS- mutant melanomas are associated with an increased risk of metastases to visceral organs and the central nervous system (CNS) [19], presenting a more aggressive clinical outcome when compared to their NRAS wild-type (WT) counterparts [20]. Their response to immunotherapy remains debated. While some studies suggest that NRAS mutations may confer improved outcomes with treatments such as high-dose interleukin-2 and immune checkpoint inhibitors targeting PD-1 or CTLA-4 [21], other investigations have reported conflicting results [22, 23], and conclusive validation in prospective studies is lacking. Resistance mechanisms, both intrinsic and acquired, frequently emerge following treatments targeting the MAPK pathway, CDK4/6 inhibitors, or immune checkpoint blockade (ICB) therapy [24–26], hampering the disease management.

### NRAS Structure Key and Conserved Domains

NRAS, along with HRAS and KRAS, belongs to the RAS oncogene family, whose discovery marked a milestone in cancer biology. The NRAS gene was the first oncogene identified in melanoma and remains the second most frequently mutated gene in this malignancy after BRAF, with a mutation prevalence of approximately 20–30% [3, 27]. This pivotal discovery dates to 1984, when activating mutations in NRAS were detected in 4 out of 30 melanoma cell lines during screenings for genes with transforming potential, firmly establishing its role as a critical oncogene [28, 29]. In contrast, mutations in other RAS isoforms, such as KRAS and HRAS, are exceedingly rare in melanoma, occurring in only 2% and 1% of cases, respectively [30].

The RAS family genes, identified between the 1960s and 1980s [31–34], encode small GTPases that play essential roles in signal transduction pathways regulating critical cellular functions, including proliferation, survival, differentiation, metabolism, and migration [35, 36]. The activity of RAS proteins is tightly regulated by their ability to cycle between an inactive GDP-bound state and an active GTP-bound state. Dysregulation of this cycle supports the oncogenic potential of RAS proteins. All RAS isoforms, including NRAS, share a highly conserved core structure essential for their function. These conserved regions are primarily responsible for nucleotide binding, GTPase activity, and interactions with downstream effectors. This conservation, particularly in the G-domain (residues 1–166), provides a degree of functional redundancy among isoforms [12]. Despite these similarities, subtle differences in their C-terminal hypervariable regions (HVRs) account for isoform-specific signaling and subcellular localization.

**Fig. 1:**
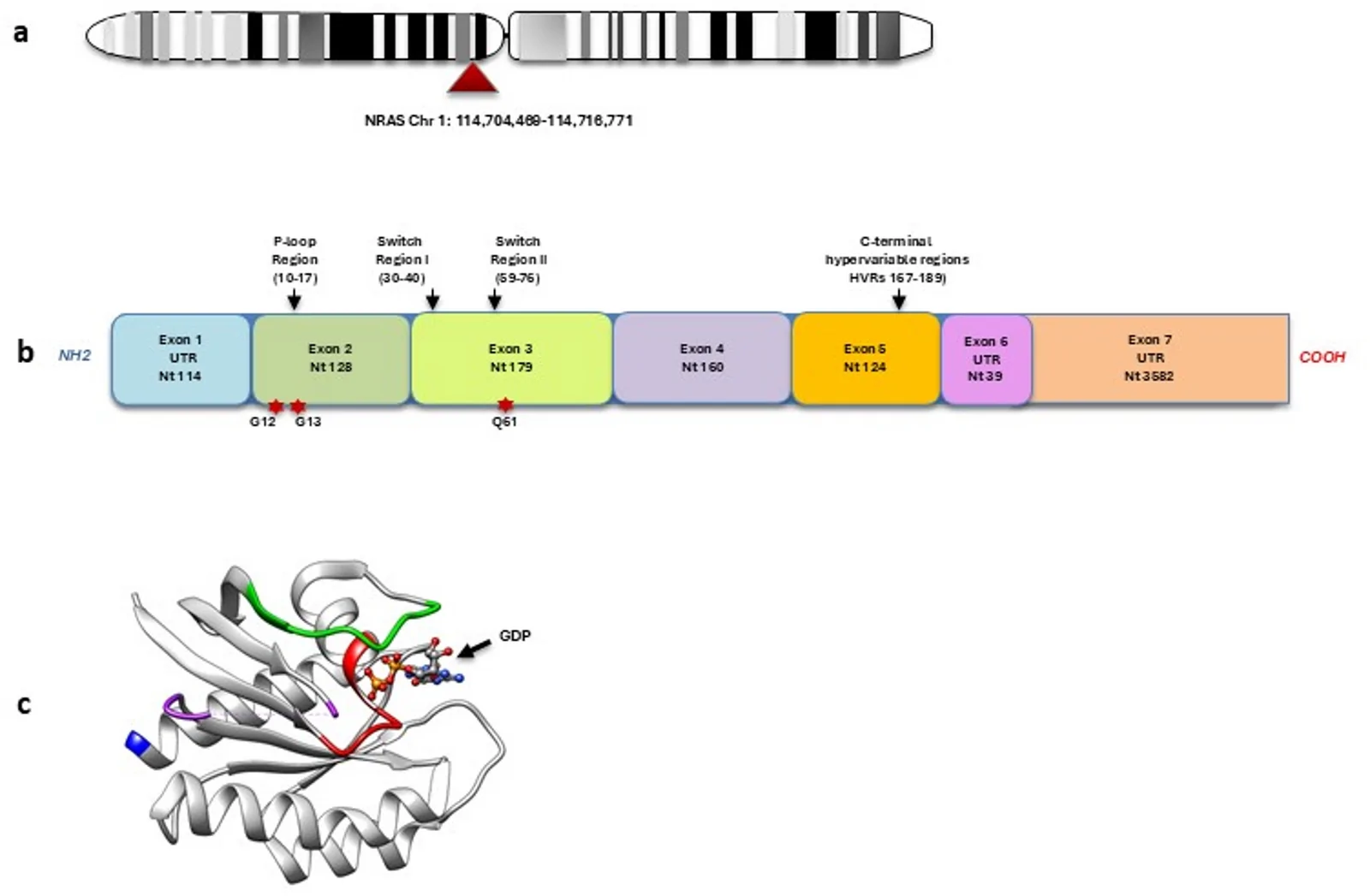
(**a**) Genomic location of the *NRAS* gene on chromosome 1; (**b**) Schematic representation of *NRAS* exons shown as colored boxes, with the number of nucleotides (Nt) indicated for each exon and highlighting the most frequent variants in malignant melanoma; (**c**) Crystal structure of the human NRAS protein, illustrating the P-loop (red), switch I region (green), switch II region (purple), hypervariable regions (HVRs, blue), and the bound guanosine 5’-diphosphate (GDP) molecule

As reported in Fig. 1, the NRAS gene maps on chromosome 1, and the structural domains of the NRAS protein include:

- The P-loop (residues 10–17): crucial for phosphate binding during GTP or GDP association.
- Switch I (residues 30–40) and Switch II (residues 59–76): involved in effector binding and nucleotide exchange.
- The C-terminal HVR: regulates subcellular localization through post-translational modifications such as farnesylation.

### Oncogenic and Functional Impact Mutations in NRAS

Oncogenic mutations in RAS genes primarily occur at codons 12, 13, or 61, which are critical for GTPase activity and nucleotide binding [37, 38]. These mutations induce structural alterations that impair the hydrolysis of GTP, locking NRAS in a constitutively active, GTP-bound state. The functional consequences of these mutations are context-dependent but generally result in persistent activation of downstream signaling pathways, including MAPK and PI3K-AKT.

- Codons 12 and 13 (P-loop): Mutations in this region disrupt the interaction of GTPase-activating proteins (GAPs) with NRAS, thereby impairing catalytic GTP hydrolysis.
- Codon 61 (Switch II): Mutations at this codon, which account for approximately 80% of NRAS mutations, directly stabilize the active GTP-bound conformation, further inhibiting GTP hydrolysis [39].

The majority of oncogenic NRAS mutations (Fig. 1), approximately 80%, occur at codon 61, with Q61K and Q61R being the most common variants [40], closely associated with aggressive tumor behavior and enhanced proliferative potential [41–43]. These mutations significantly impair the intrinsic GTPase activity of NRAS, preventing its normal cycling between active and inactive states. As a result, NRAS becomes resistant to the negative regulation typically exerted by GTPase-activating proteins (GAPs**)**, which ensures persistent and constitutive signaling. This dysregulation leads to the hyperactivation of the MAPK pathway, a key driver of cellular proliferation and survival in melanoma. Additionally, codon 61 mutations activate parallel signaling pathways, including PI3K-AKT and RAL-GDS, which contribute to therapy resistance and facilitate tumor progression. In contrast, mutations at codons 12 and 13 are less common but still play a significant role in melanomagenesis, driving disease progression by locking NRAS in its active, GTP-bound state, resulting in constitutive activation of downstream signaling pathways. This constitutive signaling fosters uncontrolled cellular proliferation, survival, and metastasis formation through key pathways such as MAPK/ERK and PI3K/AKT [44]. These mutations also impair physiological inactivation mechanisms and amplify downstream signaling outputs.

### MAPK Pathway Activation in NRAS-Mutant Melanomas

In NRAS-mutant melanomas, the MAPK signaling pathway operates predominantly through the RAS-effector CRAF, rather than BRAF, which is the primary effector in BRAF-mutant melanomas [40, 45]. This distinction underscores the unique molecular mechanisms underlying NRAS-driven melanoma. The constitutive activation of MAPK/ERK signaling contributes to a more aggressive disease phenotype, with patients exhibiting poorer overall survival (OS) compared to those with BRAF-mutant or triple wild-type melanomas (wild-type for NRAS, BRAF, and NF1) [15, 46].

### Implications for Oncogenesis

NRAS-driven cancers, including melanoma, exhibit unique biological behaviors characterized by higher metastatic potential, resistance to apoptosis, and adaptation to diverse microenvironments. Unlike BRAF mutations, which are frequently found in benign nevi, NRAS mutations are less common in benign lesions and are strongly associated with malignant transformation, particularly in older individuals [14, 15]. Despite significant advances in understanding the structural and functional dynamics of NRAS, its unique biochemical properties, such as the lack of a deep, druggable pocket, pose challenges for direct targeting. Additionally, the reliance of NRAS on protein-protein interactions for its oncogenic activity further complicates the development of effective small-molecule inhibitors [47, 48]. Consequently, therapeutic efforts have increasingly shifted toward targeting downstream effectors or exploiting synthetic lethality interactions induced by oncogenic NRAS signaling, although these strategies are still at an early stage of development within the context of melanoma.

### MEK Inhibitors in NRAS-Mutant Melanoma

MEK inhibitors (MEKi) target the RAS/RAF/MEK/ERK signaling cascade, suppressing cell proliferation and inducing apoptosis [49]. As summarized in Table 1, several clinical trials have provided significant insights into the therapeutic potential of MEK inhibitors.

**Table 1:** Clinical trials for NRAS-mutant melanoma patients. Trials included targeted therapies (MEK, RAF, ERK, and CDK4/6 inhibitors) and immunotherapies (immune checkpoint inhibitors)

Clinical Trial Number	Phase	Population	Status	Drug(s)/Notes	Reference
NCT05217303	Phase II	Advanced melanoma patients with NRAS mutation	Completed	HL-085 (Tunlametinib, MEK inhibitor)	[50]
NCT04059224	Phase II	Advanced melanoma, pretreated patients, BRAF V600 wild-type	Completed	Trametinib (MEK inhibitor) and Dabrafenib (BRAF inhibitor)	[51]
NCT03973151	Phase I	Advanced melanoma patients with NRAS mutation	Completed	HL-085 (Tunlametinib, MEK inhibitor)	[52]
NCT03932253	Phase Ia/Ib	Stage III/IV melanoma patients with NRAS-aberrant (Ia) and NRAS/NF1-mutation (Ib)	Suspended	FCN-159 (MEK1/2 inhibitor)	[53]
NCT03580382	Phase I/II	Advanced melanoma patients with NRAS mutation and BRAF/NRAS wild-type	Terminated	CDX-3379 (ERBB2 antibody) and Trametinib (MEK inhibitor)	No reference available
NCT03415126	Phase I	Patients with advanced solid tumors, including NRAS-mutated melanoma	Completed	ASN-007 (ERK1/2 inhibitor)	[54]
NCT02974725	Phase I	Patients with advanced/metastatic KRAS or BRAF-mutant NSCLC or NRAS-mutant melanoma	Terminated	LXH254 (Naporafenib, Pan-RAF TK inhibitor), LTT462 (Rineterkib, ERK1/2 inhibitor), Trametinib (MEK inhibitor)	[55]
NCT02862743	Basic Science	Patients with metastatic melanoma	Completed	Molecular characterization through blood biomarker analysis	[56]
NCT02138292	Phase Ib	Advanced melanoma, BRAF wild-type	Completed	Digoxin (cardiac glycoside with potential antitumor properties) and Trametinib (MEK inhibitor)	[57]
NCT02065063	Phase I/II	Patients with solid tumors	Completed	Trametinib (MEK inhibitor) and/or Palbociclib (CDK4/6 inhibitor)	No reference available
NCT01781572	Phase I/II	Melanoma patients with NRAS mutation	Completed	LEE011 (Ribociclib, CDK4/6 inhibitor) and MEK162 (Binimetinib, MEK inhibitor)	[58]
NCT01763164	Phase III	Unresectable/metastatic melanoma, NRAS Q61-positive	Completed	MEK162 (Binimetinib, MEK inhibitor) vs. Dacarbazine	[20]
NCT01543113	Diagnostic	Melanoma patients	Completed	Sequencing for mutations in cKIT, BRAF, and NRAS	[59]
NCT01320085	Phase II	BRAF- and NRAS-mutant metastatic melanoma	Completed	MEK162 (Binimetinib, MEK inhibitor)	[60]
NCT00866177	Phase II	BRAF- and/or NRAS-mutated melanoma	Completed	AZD6244 (Selumetinib, MEK inhibitor)	[61]
NCT00338130	Phase II	Patients with unresectable AJCC stage III/IV melanoma	Completed	Temozolomide (alkylating agent) vs. AZD6244 (Selumetinib, MEK inhibitor)	No reference available
NCT02357732	Phase I	Patients with BRAF- or NRAS-mutated metastatic melanoma	Withdrawn	Nivolumab (anti-PD1) with Dabrafenib (BRAF inhibitor) and/or Trametinib (MEK inhibitor)	No reference available

A key advancement in the management of NRAS-mutant melanoma has been the development of tunlametinib (HL-085), a next-generation, highly selective MEK1/2 inhibitor. In 2024, it received conditional authorization from the National Medical Products Administration (NMPA) of China, becoming the first and only MEK inhibitor globally approved specifically for patients with advanced NRAS-mutant melanoma [62].

The decision was supported by results from a pivotal phase II clinical study (NCT05217303) [50] involving individuals with NRAS Q61–mutated advanced melanoma who progressed after a previous systemic therapy or were intolerant to it. An earlier phase I trial (NCT03973151) [52] defined the recommended phase II dose and confirmed an acceptable safety profile, with dose-limiting toxicities consistent with those typically seen with MEK inhibitors [63].

In the phase II trial (NCT05217303) [50], including patients with advanced NRAS-mutant melanoma previously treated with multiple systemic therapies, tunlametinib showed clinically meaningful efficacy [64]: median PFS of 4.2 months (95% CI: 2.8–5.6), median OS of 13.7 months (95% CI: 10.8–17.2), objective response rate of 35.8%, and disease control rate of 71.4%. These outcomes substantially exceed those observed with binimetinib in the NEMO trial [20]. The phase III NEMO study compared binimetinib with dacarbazine in NRAS-mutant metastatic melanoma, demonstrating a significant increase in progression-free survival (PFS): median PFS of 2.8 months versus 1.5 months (HR 0.62, *p* = 0.0003) [20]. However, the study did not demonstrate a significant overall survival (OS) benefit, leading regulatory agencies to decline approval.

To date, no MEK inhibitor has been approved for NRAS-mutant melanoma in Western countries. The optimized pharmacokinetic profile of tunlametinib, including improved oral bioavailability, extended half-life, and potentially enhanced blood-brain barrier penetration, may open the way toward a wider use of a treatment showing some superior activity. Confirmatory phase III data in larger, multi-ethnic cohorts will likely be required by Western regulatory agencies before approval can be considered. This could be clinically relevant given the high incidence of CNS metastases in NRAS-mutant melanoma [19].

The benefit of tunlametinib as a monotherapy remains however moderate (median PFS 4.2 months), indicating the need for combination strategies. Future research should explore combining tunlametinib with ICIs, CDK4/6 inhibitors, or PI3K–AKT inhibitors in tumors lacking CDKN2A or PTEN.

Nevertheless, introduction of tunlametinib in clinical practice in China confirms MEK inhibition as a viable therapeutic approach and establishes a foundation for more effective combination regimens. Evidence from Western trials reinforces this need: other MEK inhibitors, such as pimasertib, have exhibited limited monotherapy activity without OS benefit [65]. Real-world analyses similarly report modest results for MEKi therapy after ICI failure, with an ORR of 18.2%, median PFS of 2.8 months, and OS of 7.1 months [66]. Innovative combination strategies, such as the pan-RAF inhibitor naporafenib (LXH254) with the anti–PD-1 antibody spartalizumab, have shown promising early activity, though these remain investigational [67].

### Optimizing Treatment Sequencing for NRAS-Mutant Melanoma

Strategic sequencing therapies may represent an alternative option for improving outcomes in NRAS-mutant melanoma. Evidence from completed clinical trials (summarized in Table 1) suggests that MEK inhibitors effectively target the hyperactivated MAPK pathway, while dual immune checkpoint blockade with ipilimumab (anti-CTLA-4) and nivolumab (anti-PD-1) enhances antitumor immunity through complementary mechanisms. The strong immunogenic potential of NRAS-mutant melanomas, driven by their high tumor mutational burden and shared neoantigens, provides a compelling rationale for integrating these approaches. However, the immunosuppressive tumor microenvironment (TME) and the low tumor-infiltrating lymphocyte (TIL) levels highlight the importance of optimizing the drug sequencing to maximize treatment efficacy. A rational approach may involve initial targeted therapy to achieve rapid tumor reduction, followed by a combination of immunotherapy to sustain long-term immune control. MAPK inhibition has been shown to enhance T-cell infiltration and activity, improving immunotherapy responses. Optimizing this treatment sequence could improve survival in patients with metastatic NRAS-mutant melanoma [68, 69].

## Histotype-Specific Patterns of NRAS Mutations in Melanoma

NRAS mutations in melanoma exhibit complex patterns influenced by histological subtype, sun exposure, and population ancestry. According to the WHO 2018 melanoma classification, NRAS mutations were initially thought to be predominantly associated with high-cumulative sun damage (CSD) melanomas [70]. However, emerging evidence from large-scale genomic studies, particularly from Asian populations, suggests a more nuanced picture.

A comprehensive study by Bai et al., analyzing 2,793 melanoma patients from a Chinese cohort, reported comparable NRAS mutation frequencies across subtypes: 9.0% in acral, 13.0% in mucosal, and 10.8% in cutaneous melanoma [71]. These findings challenge the assumption that non-CSD melanomas, including acral and mucosal subtypes, are predominantly NRAS wild type.

Population ancestry likely plays a role in the observed differences. Acral (42–65%) and mucosal (20–30%) melanomas are the predominant subtypes in Eastern countries, whereas they are rare in Caucasian populations, representing only 4–6% (acral) and < 1% (mucosal) of all melanomas [72]. The molecular landscape of these subtypes may differ meaningfully across ancestral groups, reflecting distinct etiological pathways and UV-exposure patterns. Additionally, technical variations in mutation detection methodologies may contribute to discrepancies in reported mutation frequencies.

These findings underscore the importance of population-specific genomic profiling. NRAS mutation testing should be systematically assessed across all melanoma subtypes rather than restricted by histological classification. A meta-analysis conducted by Gutiérrez-Castañeda et al., which included 3,904 patients from 17 studies, reported an overall NRAS mutation frequency of 16.4% (641 patients). NRAS mutations were particularly associated with nodular melanoma, melanomas located on the limbs, and ulcerated tumors. From the geographical point of view, mutation prevalence was higher in populations from Sweden, USA, Italy, and Spain. Interestingly, this study found no significant correlation between NRAS mutations and metastases or chronic sun exposure, which contrasts with the well-established link between BRAF mutations and intermittently sun-exposed melanomas [73].

### Independent Analysis with COSMIC database

As an integral component of this narrative review, and to further characterize the molecular landscape of NRAS-mutant malignant melanoma (MM) across different histotypes, we conducted an independent analysis of data from the Catalogue of Somatic Mutations in Cancer (COSMIC) database [https://cancer.sanger.ac.uk/cosmic] [74] (see Tables from 2 to 6). Our dataset included 2,178 melanoma patients with defined histotypes, revealing a slightly elevated NRAS mutation frequency of 24.1% (525 patients) compared to previous reports. The independent data analysis was performed using the COSMIC database, version v100 accessed on November 15th 2024). The query and data-filtering strategy were predefined as follows: skin was selected as the primary tissue, with all available sub-tissues included, and cases were subsequently categorized according to the following sub-histological classifications: superficial spreading (SSM), nodular/vertical growth (NM), acral lentiginous (ALM), desmoplastic (DM), spitzoid (SM), lentigo maligna (LMM), epithelioid (EM), blue nevus (BNM), amelanotic (AM), nevoid (NEM), and mucosal lentiginous (MLM). This approach enabled a histotype-specific assessment of NRAS mutation prevalence and co-mutation patterns, thereby providing a more precise characterization of NRAS-driven melanomagenesis.

The distribution of different histotypes among the 2,178 melanoma patients is reported in Table 2. Superficial spreading melanoma, nodular melanoma, and acral lentiginous melanoma are the most frequent histotypes, accounting for 37.6%, 34.2%, and 15.5% of 2,178 cases, respectively. All other histotypes were represented by negligible percentages.

**Table 2:** Distribution of histotypes in MM patients (*N* = 2,178) extracted from the COSMIC database

Histotype	MM Patients *N* (%)
Superficial Spreading Melanoma (SSM)	818 (37.6%)
Nodular Melanoma (NM)	744 (34.2%)
Acral Lentiginous Melanoma (ALM)	337 (15.5%)
Desmoplastic Melanoma (DM)	85 (3.9%)
Spitzoid Melanoma (SM)	61 (2.8%)
Lentigo Maligna Melanoma (LMM)	64 (2.9%)
Epithelioid Melanoma (EM)	27 (1.2%)
Blue Nevus Melanoma (BNM)	27 (1.2%)
Amelanotic Melanoma (AM)	9 (0.4%)
Nevoid Melanoma (NEM)	5 (0.2%)
Mucosal Lentiginous Melanoma (MLM)	1 (0.05%)
Total	2178

The distribution of histotypes among the 525 NRAS-mutant patients is reported in Table 3. Although superficial spreading melanoma is the most frequent histotype in the overall melanoma cohort, nodular melanoma represented the largest histological subgroup among the 525 NRAS-mutant cases (39.8%), followed by superficial spreading melanoma (33.7%) and acral lentiginous melanoma (16.6%).

**Table 3:** Distribution of histotypes in NRAS-mutant MM patients (*N* = 525; 24.1%) among the 2,178 cases from the COSMIC database

Histotype	NRAS-mutant Patients *N* (%)
Superficial Spreading Melanoma (SSM)	177 (33.7%)
Nodular Melanoma (NM)	209 (39.8%)
Acral Lentiginous Melanoma (ALM)	87 (16.6%)
Desmoplastic Melanoma (DM)	3 (0.6%)
Spitzoid Melanoma (SM)	13 (2.5%)
Lentigo Maligna Melanoma (LMM)	18 (3.4%)
Epithelioid Melanoma (EM)	14 (2.7%)
Blue Nevus Melanoma (BNM)	0 (0%)
Amelanotic Melanoma (AM)	1 (0.2%)
Nevoid Melanoma (NEM)	2 (0.4%)
Mucosal Lentiginous Melanoma (MLM)	1 (0.2%)
Total	525

The discrepancy in the mutation frequency detected by Gutiérrez-Castañeda (16.4%) and our Cosmic analysis (24.1%) may reflect methodological differences or biases in the extrapolated data. However, consistent with the findings of Gutiérrez-Castañeda et al. [73], our study revealed significant variability in NRAS mutation prevalence across histological subtypes. When normalized to the total number of cases within each histotype, nodular melanoma exhibited the highest prevalence of NRAS mutations (28.1%), followed by acral lentiginous melanoma (25.8%) and superficial spreading melanoma (21.6%). The pooled category of less represented histotypes (“Others”) showed a lower prevalence of 18.6%, as reported in Table 4. To evaluate whether these differences reflected a true biological distribution rather than variability related to unequal subgroup sizes, statistical analyses were performed across the four main histotype categories. This approach minimized potential bias associated with the marked imbalance in sample representation among subgroups.

**Table 4:** NRAS mutation frequency within each histotype

Histotype	NRAS-mutant *N* (%)	NRAS wild-type *N* (%)	Total *N*
SSM	177 (21.6%)	641 (78.4%)	818
NM	209 (28.1%)	535 (71.9%)	744
ALM	87 (25.8%)	250 (74.2%)	337
Others	52 (18.6%)	227 (81.4%)	279
Total	525 (24.1%)	1653 (75.9%)	2178

**Fig. 2:**
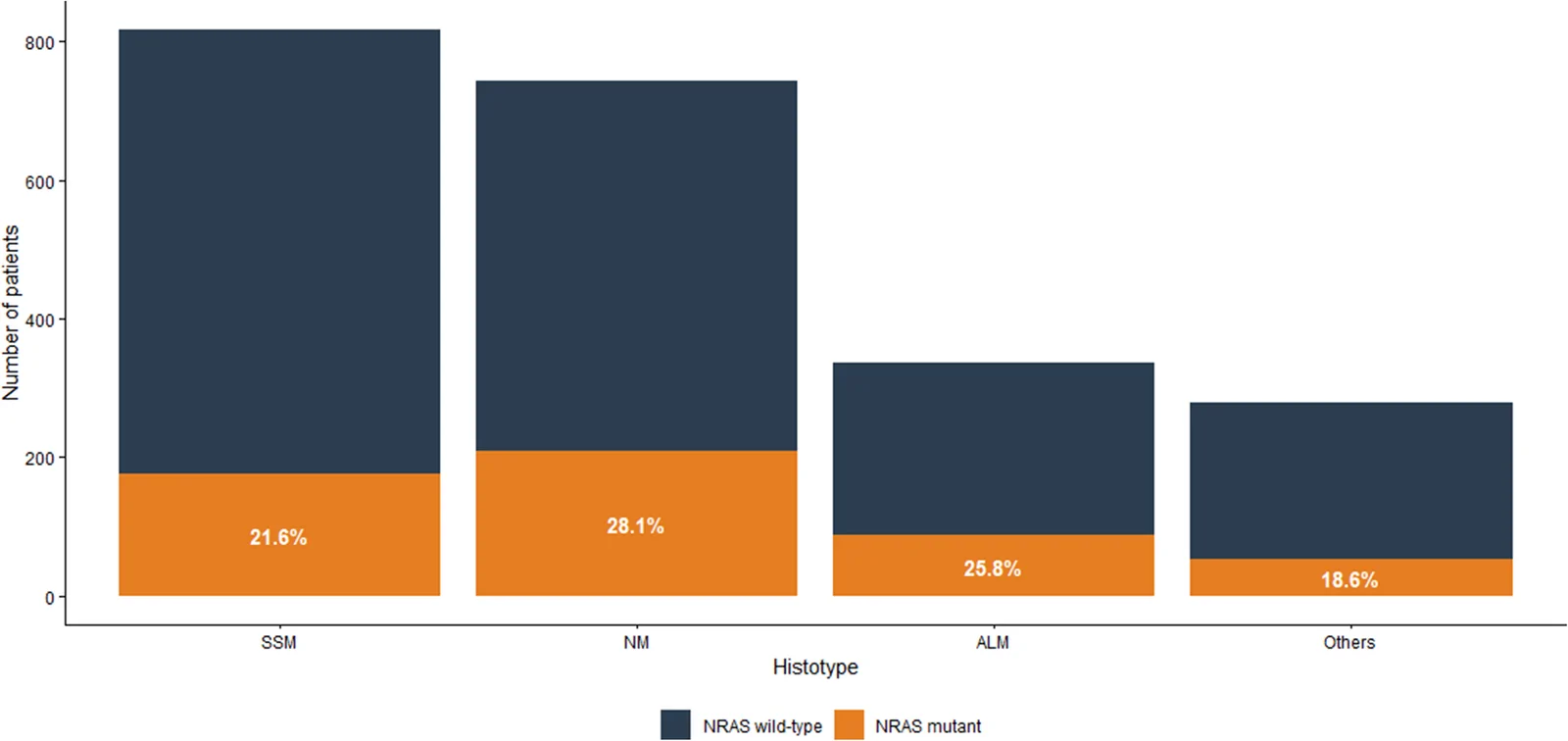
NRAS mutation prevalence across melanoma histotypes (*N* = 2178)

The prevalence of NRAS mutations across the four main MM histotypes is graphically illustrated in Fig. 2.

A chi-square test demonstrated a significant association between melanoma histotype and NRAS mutational status (χ² = 14.28, df = 3, *p* = 0.0025), indicating that the frequency of NRAS mutations differs significantly across histological subtypes. Statistical analyses were performed using R (version 4.4.0). The assumption of expected frequencies ≥ 5 in each cell was verified prior to analysis. To further identify which histotypes contributed most strongly to this association, standardized residuals (SR) were calculated, with values exceeding ± 1.96 considered statistically significant.

This analysis demonstrated that NRAS mutations are not evenly distributed among melanoma histotypes. In particular, nodular melanoma showed a significant overrepresentation of NRAS-mutant cases (SR = + 3.13), suggesting that NRAS activation may play a particularly relevant biological role in the pathogenesis of this subtype. In contrast, superficial spreading melanoma (SR = − 2.09) and the group of less common histotypes (SR = − 2.29) displayed significant underrepresentation of NRAS mutations, supporting the hypothesis that alternative oncogenic drivers, especially BRAF alterations, may predominate in these tumors [4]. Acral lentiginous melanoma did not significantly deviate from expected frequencies (SR = + 0.80), consistent with a potentially more heterogeneous molecular landscape.

Among the 525 NRAS-mutant patients, a total of 543 NRAS mutations were identified (*N* = 543), as some patients harbored more than one NRAS alteration. As reported in Table 5, the most common mutation occurred at codon Q61 (81.4%) while mutations in codons G12-13 accounted for 14.4%. Mutations in other codons were observed in a smaller fraction (4.4%), supporting previously published data [29]. The percentages related to specific NRAS gene mutations may not accurately reflect the true distribution due to the highly variable number of samples within each histotype.

**Table 5:** Distribution of NRAS mutations according to MM histotypes (COSMIC database)

Histotype	NRAS Mutation *N*	NRAS Q61 *N* (%)	NRAS G12/13 *N* (%)	NRAS others *N* (%)
Superficial Spreading Melanoma (SSM)	187	153 (81.8)	21 (11.2)	13 (7.0)
Nodular Melanoma (NM)	211	188 (89.1)	23 (10.9)	0
Acral Lentiginous Melanoma (ALM)	92	62 (67.4)	21 (22.8)	9 (9.8)
Desmoplastic Melanoma (DM)	3	3 (100)	-	-
Spitzoid Melanoma (SM)	13	11 (84.6)	2 (15.4)	-
Lentigo Maligna Melanoma (LMM)	19	11 (57.9)	7 (36.8)	1 (5.3)
Epithelioid Melanoma (EM)	14	10 (71.4)	3 (21.4)	1 (7.1)
Blue Nevus Melanoma (BNM)	0	-	-	-
Amelanotic Melanoma (AM)	1	1 (100)	-	-
Nevoid Melanoma (NEM)	2	2 (100)	-	-
Mucosal Lentiginous Melanoma (MLM)	1	-	1(100)	-
Total	543	441 (81.4)	78 (14.4)	24 (4.4)

The strong NRAS mutation enrichment in nodular melanoma aligns with its aggressive clinical phenotype, characterized by rapid vertical growth, early metastatic spread, and poor prognosis [75]. This molecular-pathological correlation likely reflects the biological consequences of constitutive MAPK pathway activation, including enhanced proliferation, invasion, and survival signaling. The underrepresentation in superficial spreading melanoma is consistent with the known predominance of BRAF mutations in this subtype, while the heterogeneous pattern in acral melanoma underscores the need for comprehensive genomic profiling beyond single-gene testing [17].

These findings may have potential clinical implications, although they should be considered hypothesis-generating. Nodular melanoma demonstrated a relatively higher prevalence of NRAS mutations in our cohort, suggesting that histological subtype could contribute to hypothesis-generating strategies for molecular testing prioritization. However, these observations should be interpreted cautiously given the retrospective and exploratory nature of this database analysis. Histological subtypes may contribute to molecular testing strategies and therapeutic decision-making, with treatment algorithms potentially requiring histotype-specific stratification, particularly for MEK inhibitor therapy selection. Risk stratification and surveillance protocols may also benefit from integrating both histological subtype and mutational status, given their possible combined prognostic implications [76–78]. In summary, our analysis reveals that histological classification provides valuable information about the likelihood of NRAS mutations, enabling more efficient molecular testing strategies and personalized therapeutic approaches in melanoma management. Prospective validation in independent, clinically annotated cohorts will be essential to determine whether histological subtype and NRAS mutational status provide complementary information for prognostic stratification, disease surveillance, and treatment selection.

### Coexistence of Mutations in NRAS and Other Genes

Analysis of 2,178 melanoma cases in the COSMIC database revealed that among 525 NRAS-mutant patients, 28 cases exhibited double NRAS mutations, suggesting intratumoral heterogeneity with distinct subclonal populations. Co-occurring mutations included: BRAF (10 V600 cases; 5.9%), CDKN2A (5.7%), TP53 (2.5%), RAC1 (2.1%), NF1 (1.9%), and PTEN with KIT (1.7%). Less frequent alterations involved IDH1, CTNNB1, TERT, JAK1/2/3, GNAQ/GNA11, PIK3CA, and MITF. The distribution of co-occurring mutations across the four main melanoma histotypes is reported in Table 6.

**Table 6:** Co-occurring mutations in NRAS-mutant MM patients (*N* = 525) across histological subtypes extracted from the COSMIC database

Gene	Total *N* (%)	SSM *N* (%)	NM *N* (%)	ALM *N* (%)	Others *N* (%)
CDKN2A	30 (5.7%)	17 (9.6%)	11 (5.3%)	-	2 (3.8%)
BRAF	31 (5.9%)	13 (7.3%)	12 (5.7%)	2 (2.3%)	4 (7.7%)
Double NRAS	28 (5.3%)	14 (7.9%)	8 (3.8%)	5 (5.7%)	1 (1.9%)
TP53	13 (2.5%)	7 (4.0%)	5 (2.4%)	-	1 (1.9%)
RAC1	11 (2.1%)	5 (2.8%)	5 (2.4%)	-	1 (1.9%)
NF1	10 (1.9%)	5 (2.8%)	4 (1.9%)	-	1 (1.9%)
PTEN	9 (1.7%)	5 (2.8%)	3 (1.4%)	-	1 (1.9%)
KIT	9 (1.7%)	5 (2.8%)	3 (1.4%)	-	1 (1.9%)
IDH1	8 (1.5%)	6 (3.4%)	2 (1.0%)	-	-
CTNNB1	6 (1.1%)	1 (0.6%)	3 (1.4%)	1 (1.1%)	1 (1.9%)
JAK1	5 (1.0%)	3 (1.7%)	2 (1.0%)	-	-
GNAQ	5 (1.0%)	3 (1.7%)	2 (1.0%)	-	-
TERT	4 (0.8%)	3 (1.7%)	1 (0.5%)	-	-
PIK3CA	4 (0.8%)	-	2 (1.0%)	-	2 (3.8%)
BAP1	3 (0.6%)	1 (0.6%)	2 (1.0%)	-	-
JAK3	2 (0.4%)	1 (0.6%)	1 (0.5%)	-	-
GNA11	2 (0.4%)	1 (0.6%)	1 (0.5%)	-	-
MITF	2 (0.4%)	-	2 (1.0%)	-	-
JAK2	1 (0.2%)	-	1 (0.5%)	-	-

Percentages are calculated on the total NRAS-mutant patients within each histotype (SSM *N* = 177; NM, *N* = 209; ALM, *N* = 87; Others, *N* = 52). Others include Desmoplastic Melanoma (DM, *N* = 3), Spitzoid Melanoma (SM, *N* = 13), Lentigo Maligna Melanoma (LMM, *N* = 18), Epithelioid Melanoma (EM, *N* = 14), Amelanotic Melanoma (AM, *N* = 1), Nevoid Melanoma (NEM, *N* = 2), and Mucosal Lentiginous Melanoma (MLM, *N* = 1).

### Functional Implications of Co-Mutations in NRAS-Mutant Melanoma

The coexistence of NRAS mutations with alterations in other cancer-related genes defines molecularly distinct melanoma subtypes, each with unique therapeutic vulnerabilities and clinical outcomes. Among the 525 NRAS-mutant patients, co-occurring mutations are reported in Table 6 and provide actionable information for treatment selection, resistance prediction and prognosis.

- **NRAS-BRAF Co-Mutations (***N***= 31**,** 5.9%)**: Although generally considered mutually exclusive, these co-mutations likely reflect intratumoral heterogeneity, with spatially distinct clonal populations [79]. Combined MAPK pathway inhibition may be required, though clinical data remain limited.
- **NRAS-CDKN2A Co-Mutations (*N*= 30**,** 5.7%):** Concurrent NRAS activation and CDKN2A loss (encoding both p16^INK4a and p14^ARF) generate a highly aggressive melanoma phenotype. NRAS activation drives RAS-MAPK-mediated proliferation, while CDKN2A loss simultaneously impairs p16^INK4a-mediated cell cycle control and p14^ARF-dependent p53 tumor suppression. This synergy accelerates proliferation, enhances resistance to apoptosis, and facilitates early metastatic dissemination. This co-mutation pattern has prompted clinical investigation of combined CDK4/6 and MEK inhibition, with affected patients exhibiting shorter time to recurrence and reduced overall survival compared to single-mutation cases [58].
- **NRAS-TP53 Co-Mutations (*N* = 13**,** 2.5%):** These alterations affect two complementary pathways. NRAS activation drives the MAPK proliferative cascade, while TP53 loss, the “guardian of the genome” [80], promotes genomic instability and impairs apoptosis. Their combination may synergistically enhance tumor aggressiveness and disease progression [81].

Co-occurrence of NRAS mutations with PTEN (9, 1.7%), RAC1 (11, 2.1%), NF1 (10, 1.9%), CTNNB1 (6, 1.1%), and JAK1/2/3 (8, 1.5%) generates melanomas with concurrent disruption of proliferative, migratory, and immune-regulatory pathways. These co-mutations enforce NRAS-driven oncogenic signaling, through dual MAPK/PI3K activation, enhanced invasion, aberrant transcriptional reprogramming, and impaired interferon response, resulting in more aggressive disease and variable therapeutic vulnerabilities [82–85].

### Limitations of the COSMIC Database

Several limitations of the COSMIC database should be considered when interpreting the present findings. A major challenge arises from the substantial heterogeneity of the data sources incorporated into COSMIC, which include published literature, genome-wide screens, and large-scale consortia. This heterogeneity may introduce reporting biases toward well-studied or clinically annotated cases, while the accuracy and consistency of histotype classification ultimately depend on the quality and completeness of the underlying primary data. In addition, COSMIC often lacks detailed clinical annotations, including disease stage, treatment history, and patient outcomes, thereby limiting the possibility of directly correlating mutational profiles with clinical variables. Finally, as a retrospective, aggregated repository, COSMIC does not allow independent verification of individual-level data quality or definitive resolution of potential duplicate entries resulting from overlapping studies.

Anyway, these limitations do not diminish the value of the present analysis but rather define the appropriate context in which its findings should be interpreted. Despite the inherent heterogeneity of the underlying dataset, the large number of cases available in COSMIC enabled us to examine NRAS mutational patterns across multiple melanoma histotypes within a common analytical framework, providing a broader perspective than would generally be achievable in single-institution or smaller clinical cohorts. The identification of significant histotype-specific differences in NRAS mutation frequency, together with the characterization of recurrent NRAS mutational hotspots and co-mutation patterns, provides relevant evidence supporting the non-uniform distribution of NRAS alterations across melanoma subtypes. These findings therefore offer a useful exploratory framework for generating biologically and clinically testable hypotheses and for guiding future studies based on prospectively collected, clinically annotated cohorts. Collectively, the present analysis should be regarded not as a substitute for prospective clinical validation, but as a complementary data-driven approach that contributes to a more comprehensive understanding of the molecular heterogeneity of NRAS-mutant melanoma.

## Target and Immunotherapy in NRAS-Mutant Melanoma

ICIs have transformed the therapeutic landscape for advanced melanoma by blocking inhibitory pathways that limit T-cell activation, thereby enhancing immune responses. However, cancer cells can exploit these checkpoints to evade immune surveillance, making their inhibition a promising strategy for immunotherapy [50]. Monoclonal antibodies targeting programmed cell death protein 1 (PD-1), its ligand (PD-L1), and cytotoxic T- lymphocyte-associated protein 4 (CTLA-4) demonstrated robust activation of the adaptive immune system, restoring tumor recognition and destruction by host immune defense [86]. Despite these advances, NRAS-mutant melanoma presents distinct challenges in terms of responsiveness to ICI-based therapies.

The impact of NRAS mutations on the outcome of patients treated with ICI remains controversial. Some studies have found that patients with NRAS mutations present a shorter median survival than those without the NRAS mutations [25, 87]. In this regard, NRAS-mutant melanoma was reported to be associated with an immunosuppressive TME and with activation of intrinsic resistance mechanisms that contribute to poorer clinical outcomes [88]. This latter study on 912 patients revealed that NRAS-mutant melanomas were associated with significantly lower TILs, inducing a change toward a more immunosuppressive TME, potentially limiting the ability of the immune system to recognize and eliminate tumor cells. The diminished presence of TILs may further contribute to immune evasion mechanisms and reduce the effectiveness of immunotherapies targeting NRAS-mutant melanoma.

Additional studies have reported reduced responses to anti-PD-1 monotherapy in NRAS-mutant melanoma. Zhou et al., in an analysis of four clinical trials involving patients with advanced melanoma, observed significantly lower response rates, shorter progression-free survival (PFS), and decreased overall survival (OS) in NRAS-mutant patients compared to NRAS wild-type individuals, particularly in non-cutaneous melanoma subtypes [23]. Similarly, van Not et al., analyzed a cohort of 2,528 advanced melanoma patients − 1,764 treated with anti-PD-1 therapy and 759 with ipilimumab-nivolumab, demonstrating that NRAS-mutant individuals had poorer responses, with markedly worse PFS and OS compared to those with BRAF-mutant melanoma undergoing targeted therapy [89].

To further support the lack of clarity for the NRAS mutational status on responsiveness to ICI-based regimen, other authors indicated a comparable outcome in terms of PFS and OS for patients with NRAS-mutant and NRAS-wild type melanoma undergoing ICI treatment [90], while others even reported better responses in patients with NRAS-mutant melanoma [21]. In this latter report, NRAS-mutant melanomas were found to present a higher rate of PD-L1 expression than wild-type melanomas before ICI administration. Overall, some NRAS-mutant melanoma patients exhibit responses to ICIs. Johnson et al. reported that 28% of patients with NRAS-mutant melanoma achieved complete or partial responses to first-line immunotherapy, particularly with anti-PD-1/PD-L1 agents, possibly due to higher PD-L1 expression in these tumors [21]. A recent meta-analysis by Jaeger et al. further supports this, suggesting that NRAS-mutant melanomas may be more likely to respond to ICIs compared to NRAS wild-type cases [91]. The immunogenicity of NRAS-mutant melanoma is linked to its mutational burden and shared neoantigens [3, 92]. NRAS mutations, particularly Q61 hotspot variants, can generate neo-antigens capable of eliciting immune responses. Etxeberria et al. identified a highly immunogenic neoantigen derived from HLA-A*01:01 and RAS.Q61K, which is relevant to approximately 3% of melanoma patients and demonstrates natural immunogenicity through specific tumor-infiltrating lymphocyte reactivity [93, 94]. Emerging strategies such as neoadjuvant immunotherapy may provide additional benefits for NRAS-mutant melanoma patients. Ebert et al. demonstrated that MAPK pathway inhibition via MEK inhibitors can enhance T-cell-mediated antitumor responses when combined with PD-L1 blockade [95]. MEK inhibition preserved CD8 + T-cell infiltration and function, leading to durable tumor regression. Additionally, high sensitivity circulating tumor DNA assays and refined pathological analyses of surgical specimens may help optimize immunotherapeutic strategies by identifying predictive biomarkers of response [96]. These findings evidence the potential for T-cell-based immunotherapies targeting shared neoantigens.

Altogether, although data from large multicenter cohorts were mainly retrospective, the occurrence of NRAS mutations was tendentially associated with less favorable survival [13, 21, 23, 25, 89, 90]. In the only large recent prospective study, NRAS mutational status was demonstrated to have no impact on PFS and OS among patients treated with anti-PD1-based ICI. Moreover, a similar response rate was seen in NRAS wild-type and NRAS-mutant melanoma patients. In the same study, tumor PD-L1 expression did not correlate with NRAS mutational status [26].

## Conclusions

This review dissects the pivotal role of NRAS mutations in melanoma, highlighting their unique molecular characteristics, histotype-specific distribution, and clinical implications. NRAS mutations, predominantly occurring at codon 61, drive aggressive tumor behavior by constitutively activating key signaling pathways, including MAPK and PI3K-AKT. These mutations are particularly prevalent in nodular melanomas and melanomas associated with high cumulative sun damage, although they are also detected across diverse histological subtypes, challenging traditional classifications. Clinically, NRAS-mutant melanomas pose significant therapeutic challenges due to the lack of direct inhibitors, limited efficacy of downstream pathway-targeted therapies, and an immunosuppressive tumor microenvironment. Nevertheless, the intrinsic immunogenicity of NRAS-mutant tumors offers encouraging prospects for immunotherapy, as some patients benefit from immune checkpoint inhibitors. Clinical outcomes, however, remain suboptimal, emphasizing the importance of better understanding the molecular pathways that govern NRAS-driven tumorigenesis and resistance.

Our analysis of COSMIC data complements the existing literature by revealing significant histotype-specific variability in NRAS mutation prevalence. Given the retrospective and heterogeneous nature of the COSMIC dataset and the limitations in clinical annotation, these findings should be considered exploratory and hypothesis-generating rather than as evidence for immediate changes in clinical practice. Nevertheless, the observed histotype-specific distribution of NRAS alterations provides a useful framework for investigating whether histological and molecular features may provide complementary information for future risk stratification and therapeutic studies. Prospective validation in well-characterized, clinically annotated cohorts will be essential to determine the clinical relevance of these associations.

Future efforts should focus on developing innovative strategies to target NRAS-mutant melanoma, including combination therapies that target compensatory pathway dependencies or modulate the tumor microenvironment. Emerging concepts such as synthetic lethality, in which oncogenic NRAS creates exploitable vulnerabilities in compensatory survival pathways, provide a promising framework that warrants systematic investigation in melanoma-specific models. Advances in precision medicine, supported by integrative genomic and transcriptomic analyses, hold the potential to transform the management of NRAS-mutant melanoma. Addressing the current gaps in understanding NRAS-driven tumorigenesis and resistance mechanisms will be critical for improving outcomes in this challenging subset of melanoma patients.

## Key References

- Ascierto PA, Schadendorf D, Berking C, Agarwala SS, Van Herpen CM, Queirolo P, et al. MEK162 for patients with advanced melanoma harbouring NRAS or Val600 BRAF mutations: a non-randomised, open-label phase 2 study. The Lancet Oncology [Internet]. 2013 [cited 2025 Nov 21];14:249–56. https://doi.org/10.1016/S1470-2045(13)70024-X.
  - ⚬ Of outstanding importance: it was among the first prospective trials to demonstrate the clinical efficacy of MEK inhibition in NRAS-mutant melanoma. It confirmed the MAPK pathway as a viable therapeutic target and provided the rationale for the subsequent development of binimetinib in Phase III trials, as well as supporting future combination and sequencing strategies with immunotherapy.
- Dummer R, Schadendorf D, Ascierto PA, Arance A, Dutriaux C, Di Giacomo AM, et al. Binimetinib versus dacarbazine in patients with advanced NRAS-mutant melanoma (NEMO): a multicentre, open-label, randomised, phase 3 trial. The Lancet Oncology [Internet]. 2017 [cited 2025 Nov 21];18:435–45. https://doi.org/10.1016/S1470-2045(17)30180-8.
  - ⚬ Of outstanding importance: this study is considered a landmark reference because it was the first phase III trial conducted exclusively in patients with NRAS-mutant melanoma, demonstrating the clinical activity of the MEK inhibitor binimetinib and establishing MEK inhibition as a therapeutic backbone for this molecular subtype. The NEMO trial therefore provides the strongest clinical evidence supporting targeted treatment strategies in NRAS-mutant melanoma.
- Jaeger ZJ, Raval NS, Maverakis NKA, Chen DY, Ansstas G, Hardi A, et al. Objective response to immune checkpoint inhibitor therapy in NRAS-mutant melanoma: A systematic review and meta-analysis. Front Med [Internet]. 2023 [cited 2025 Nov 21]; 10:1090737. https://doi.org/10.3389/fmed.2023.1090737.
  - ⚬ Summarizes current evidence on the efficacy of immune checkpoint inhibitors in NRAS-mutant melanoma through systematic review and meta-analysis.
- Zaremba A, Mohr P, Gutzmer R, Meier F, Pföhler C, Weichenthal M, et al. Immune checkpoint inhibition in patients with NRAS mutated and NRAS wild type melanoma: a multicenter Dermatologic Cooperative Oncology Group study on 637 patients from the prospective skin cancer registry ADOREG. European Journal of Cancer [Internet]. 2023 [cited 2025 Nov 21]; 188:140–51. https://doi.org/10.1016/j.ejca.2023.04.008.
  - ⚬ This large multicenter study evaluated the effectiveness of immune checkpoint inhibitors in patients with NRAS-mutant melanoma and provides robust real-world evidence on treatment outcomes. Its findings contribute significantly to the current understanding of the clinical impact of NRAS mutations in the immunotherapy era.
- Janku F, Kim TM, Iyer G, Spreafico A, Elez E, De Jonge M, et al. First-in-human study of naporafenib (LXH254) with or without spartalizumab in adult patients with advanced solid tumors harboring MAPK signaling pathway alterations. European Journal of Cancer [Internet]. 2024 [cited 2025 Nov 21];196:113458. https://doi.org/10.1016/j.ejca.2023.113458.
  - ⚬ This first-in-human study investigated the pan-RAF inhibitor naporafenib, alone or in combination with immunotherapy, in tumors harboring MAPK pathway alterations. The findings highlight emerging therapeutic approaches that may help overcome current limitations in the treatment of NRAS-driven melanoma.
- Wei X, Zou Z, Zhang W, Fang M, Zhang X, Luo Z, et al. A phase II study of efficacy and safety of the MEK inhibitor tunlametinib in patients with advanced NRAS-mutant melanoma. European Journal of Cancer [Internet]. 2024 [cited 2025 Nov 21];202:114008. https://doi.org/10.1016/j.ejca.2024.114008.
  - ⚬ This phase II study reported promising clinical activity and manageable toxicity of the novel MEK inhibitor tunlametinib in advanced NRAS-mutant melanoma, representing one of the most relevant recent advances in targeted therapy for this molecular subgroup.
- Liang Y, Maeda O, Nishida K, Chretien B, Ando Y. Genomic profiles of patients with skin melanoma in the era of immune checkpoint inhibitors. Cancer Science [Internet]. 2025 [cited 2025 Nov 21]; 116:1107–14. https://doi.org/10.1111/cas.16338.
  - ⚬ Provides an updated overview of the genomic landscape of melanoma and the clinical relevance of NRAS mutations in patients treated with immune checkpoint inhibitors.
- Janku F, Kim TM, Iyer G, Spreafico A, Elez E, De Jonge M, et al. First-in-human study of naporafenib (LXH254) with or without spartalizumab in adult patients with advanced solid tumors harboring MAPK signaling pathway alterations. European Journal of Cancer [Internet]. 2024 [cited 2025 Nov 21];196:113458. https://doi.org/10.1016/j.ejca.2023.113458.
  - ⚬ This first-in-human study investigated the pan-RAF inhibitor naporafenib, alone or in combination with immunotherapy, in tumors harboring MAPK pathway alterations. The findings highlight emerging therapeutic approaches that may help overcome current limitations in the treatment of NRAS-driven melanoma.

## Data Availability

Data presented in the paper are an independent analysis from the Catalog of Somatic Mutation in Cancer (COSMIC v100) database https:// cancer.sanger.ac.uk/cosmic.
